# Simple, fast, and accurate methodology for quantitative analysis using Fourier transform infrared spectroscopy, with bio-hybrid fuel cell examples

**DOI:** 10.1016/j.mex.2016.02.002

**Published:** 2016-02-21

**Authors:** David M. Mackie, Justin P. Jahnke, Marcus S. Benyamin, James J. Sumner

**Affiliations:** U.S. Army Research Laboratory, 2800 Powder Mill Road, Adelphi, MD, USA

**Keywords:** Fourier transform infrared, FTIR, Quantitative analysis, Methodology, Fuel cell

## Abstract

The standard methodologies for quantitative analysis (QA) of mixtures using Fourier transform infrared (FTIR) instruments have evolved until they are now more complicated than necessary for many users’ purposes. We present a simpler methodology, suitable for widespread adoption of FTIR QA as a standard laboratory technique across disciplines by occasional users.

• Algorithm is straightforward and intuitive, yet it is also fast, accurate, and robust.
• Relies on component spectra, minimization of errors, and local adaptive mesh refinement.
• Tested successfully on real mixtures of up to nine components.

We show that our methodology is robust to challenging experimental conditions such as similar substances, component percentages differing by three orders of magnitude, and imperfect (noisy) spectra. As examples, we analyze biological, chemical, and physical aspects of bio-hybrid fuel cells.

## Graphical abstract

**Figure:**
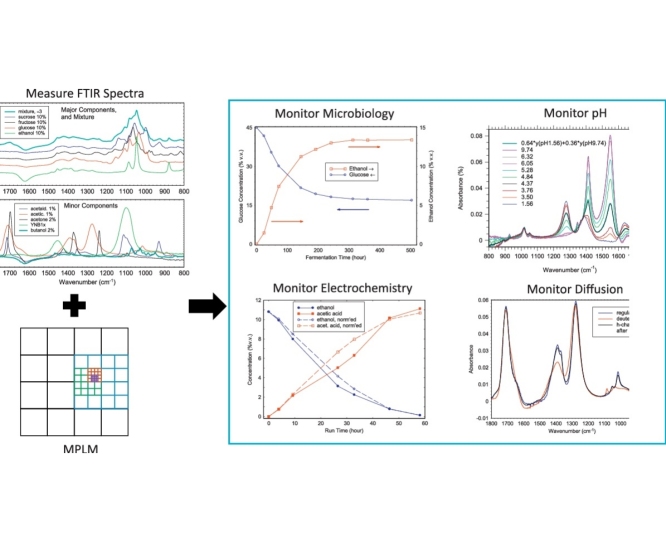

## Method details

Fourier transform infrared (FTIR) spectrometers have become a common feature of most laboratories, and are used in a wide variety of research [1], [2], [3], [4], [5], [6], [7], [8], [9], [10]. Over the last 50 years, quantitative analysis (QA) of mixtures using FTIR has progressed from matching peaks at a few wavenumbers to matching entire spectra. In the process, FTIR QA has become conjoined with specialized chemometrics software (usually commercial) that employs sophisticated linear systems theory to direct the entire course of experiments. The software first prescribes known mixtures to measure, based upon the concentration ranges of the known substances in the samples (*i.e.*, unknown mixtures) to be tested. The software uses the spectra of the specified mixtures to construct a QA model. It can then use the QA model to analyze many samples, quickly and accurately (for samples that conform to the limits of the QA model).

While this standard methodology for FTIR QA works quite well under many circumstances, it has some serious drawbacks. For occasional users, the QA software may not be available. Even if available, it can be a mysterious black box to the uninitiated. The standard linear systems methodology is needlessly complicated for many analyses. Lastly, extra QA models may be required for mixtures in which the concentrations of components vary widely, or if another component must be added to the mixture. Each change in the experiment often requires another QA model (or several more). These drawbacks may deter users, reducing adoption of FTIR QA as a standard cross-disciplinary laboratory technique.

We present a much simpler FTIR QA methodology, which we have developed to analyze the component percentages in mixtures of known substances. (We have tested mixtures with up to nine components.) The algorithm is straightforward and intuitive, yet it is also fast and accurate.

## Explanation of methodology

As usual in FTIR spectroscopy, we start with Beer's Law, which states that the absorbance spectrum of a mixture, $A_{m}^{\text{Mixture}}$, is the linear sum of the absorbance spectra of the *N* components, $A_{n,m}^{\text{Component}}$, weighted by the component concentrations, *C*_*n*_:

(1) $$A_{m}^{\text{Mixture}}=\sum_{n=1}^{N}C_{n}A_{n,m}^{\text{Component}}$$

where the *m* index runs over the *M* measured wavenumbers, the *n* index runs over the *N* components of the mixture, and *M* ≫ *N*. Strictly speaking, this law is only valid in the limit as the absorbance goes to zero. However, it has been found to be a very good approximation over a wide range of absorbances. In practice, one can usually dilute a highly-absorbing mixture until Beer's Law applies (or use a thinner sample, for transmission FTIR).

The challenge is to find *C*_*n*_ which satisfy (1). More realistically, since there is always noise in the measurements of $A_{n,m}^{\text{Component}}$ and (even more so) $A_{m}^{\text{Mixture}}$, one seeks to find *C*_*n*_ which satisfy (1) as nearly as possible. Thus, one seeks to minimize the root-mean-square error, *ɛ*_*RMS*_, with respect to the *C*_*n*_, where:

(2) $$\varepsilon_{RMS}=\frac{1}{M}\sqrt{\sum_{m=1}^{M}{\left(A_{m}^{\text{Mixture}}-\sum_{n=1}^{N}C_{n}A_{n,m}^{\text{Component}}\right)}^{2}}$$

The current standard treatment would at this point take the partial derivatives of (2) with respect to the *C*_*n*_, obtaining a set of *M* equations over-determined for the *C*_*n*_, which would then be solved through singular value decomposition (SVD). Alternative sophisticated methodologies might be used instead (or in addition), such as principal components analysis (PCA) or partial least squares (PLS). However, given current computing speeds (even for inexpensive laptop computers), one can profitably go all the way back to the “stone age” of chemometrics (*c.* 1900 *A.D.*) and apply the simple, brute force, methodology that was used when only a few peak heights were measured. That is, one can directly calculate *ɛ*_*RMS*_ using (2) for every possible combination of *C*_*n*_, to whatever precision is desired. We will refer to this as the “single mesh method” (SMM).

For mixtures of only 2 components, we have found the SMM quite satisfactory, giving accurate results to precisions of 0.1% in about 15 min. All calculations were done with (non-compiled) Microsoft Visual Basic 2010, on a laptop with a 2.66 GHz Intel Core2 Duo CPU and 4 GB RAM, running under 64-bit Windows 7. The SMM calculation time can be significantly reduced if *a priori* knowledge is available for rough estimates (as is often the case). Nevertheless, the solution time for the SMM increases quickly as one increases *N*. Therefore we developed a multi-pass method, one step up in sophistication from the brute force SMM. It was inspired by the local adaptive mesh refinement (LAMR) algorithms of partial differential equations solvers [11].

Our multi-pass “LAMR” method (MPLM) starts out as the SMM, except with a coarse *C*_*n*_ mesh. The key principle behind the MPLM is as follows: At each pass, the mesh step-size only needs to be sufficient to ensure that the resulting *C*_*n*_ values (*i.e.*, the mesh values which minimize *ɛ*_*RMS*_ in that pass) are in the vicinities of the *C*_*n*_ values which will globally minimize *ɛ*_*RMS*_. Our experience has been that we can set the first-pass mesh step-size, *s*_1_, to 10% (bounded from 0% to 50%) and not miss the global minimum. Even for mixtures of 9 components, plus a background component, this initial coarse calculation proceeds quickly (<5 min).

**Fig. 1:**
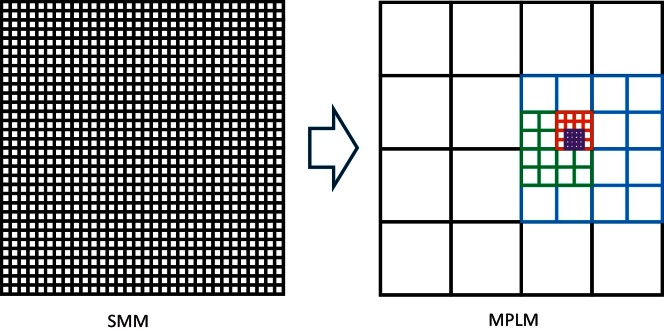
Simplified illustration, for only 2 components, of improving on the simple mesh method (SMM) with the multi-pass “local adaptive mesh refinement” method (MPLM). Five MPLM passes are shown, with the mesh halved after each pass. The outer corners of the black grid represent the initial ranges for the first pass. Each pass gets closer to the optimum fit, located somewhere within the purple grid. For 2 components, as shown, MPLM would achieve twice the accuracy for 1/10 the effort. For 9 components, the effort would be reduced by 5 million. (Actual parameters used were different, and the speed-up achieved was even greater; see text.)

In subsequent passes, new upper and lower boundaries are set based upon the previous pass, and the mesh step-size, *s*_*i*_, is reduced appropriately. This is shown schematically in Fig. 1. For simplicity, Fig. 1 shows each pass's boundaries “backed off” from the previous pass's result by one entire step-size (in every direction) of the previous pass's mesh. In our program, the user can actually choose the amount of “backing off,” *B*. In the results presented here, we used *B* = 0.8 steps, in every direction. Also, Fig. 1 shows the step-size reduced by a factor of 2 for simplicity, but our program allows the user to choose the step-size reduction. In the results presented here, the mesh step-size reduction, r, was chosen to be 4, so that *s*_*i+1*_ = *s*_i_*B/*r* = *s*_*i*_/5. Lastly, Fig. 1 shows the same mesh step-sizes used for both components, but our program does not have that limitation.

To accommodate a variety of choices for *s*_1_, *B*, and *r*, our MPLM program is coded for up to 9 passes. For our chosen parameters, 9 passes yields a potential step-size reduction from 10% (at most) for pass 1 to 10%/5^8^ = 0.000026% for step 9. Since such accuracy is unnecessary (and not merited by our measurements), our MPLM program saves time by not looping on components once the step-size < 0.01%.

In principle, the MPLM is more prone than the SMM to finding a local minimum rather than a global minimum. This was especially a concern with many components, since the convergence of many-variable minimizations has been an active area of study for at least 50 years. However, in practice we have not found this to be a problem with our MPLM. Probably this is because our component FTIR spectra are effectively orthogonal. On the occasions when we had similar component spectra, and we wanted to ensure precise final results for the *C*_*n*_ values, we simply re-ran the MPLM calculations, using a smaller value for *s*_1_. For those re-calculations, in the spirit of MPLM, we also restricted first-pass *C*_*n*_ boundaries somewhat, based on the first MPLM calculation's result, to save time on the re-calculation.

The MPLM is no more accurate than the SMM, but is much faster. For *N* components, and using the typical mesh and program parameters just mentioned, the number of computations of *ɛ*_*RMS*_ using (2) is reduced by *η* for the same final precision, where

(3) $$\eta =\frac{{\left(\text{100/0.01}\right)}^{N}}{9\times 10^{N}}\approx \frac{10^{5N}}{10\times 10^{N}}=10^{4N-1}$$

For 9 components, the reduction in computations of *ɛ*_*RMS*_ for MPLM compared to SMM can be a factor of 10^35^. Having only a few components and good *a priori* knowledge of concentrations can reduce this extraordinary advantage of MPLM over SMM. For most cases of interest, though, MPLM will be much faster than SMM, for the same desired final precision.

The main part of our program, containing the core algorithms, is available in the Supplemental Materials, along with screen shots of the interface. The full Visual Basic project files are rather large, and can quickly go out of date. The latest project files will be made available by the corresponding author, upon request.

For the measurements discussed here, because our samples were all aqueous, three additional steps were taken that are not generally necessary for fitting FTIR spectra using the MPLM. First, we used water (rather than air) as the background. This eliminated the water component from every mixture (while implicitly accounting for it). Second, we re-measured the water background just before each component and sample measurement. This increased the signal to noise of the spectra. Third, we measured component spectra at various concentrations. Then, we fit the sample spectra using component spectra measured at concentrations roughly matching those expected in the sample, to compensate for slight deviations from Beer's law due to water-component interactions and detector saturation. If any of our initial guesses for component concentrations were far (>15%) from the results of the fit, we re-fit using more-appropriate component spectra. Except in extreme cases this refitting did not change the fit results significantly, but it did increase the accuracy in test samples, so we retained this technique in our methodology.

## Verifications of methodology

Here we show that our methodology is robust under challenging experimental conditions such as components with similar spectra, component percentages differing by orders of magnitude, and imperfect (noisy) spectra. We also show that it provides a warning if a mixture contains unknown components.

### Verification with artificial mixture

**Fig. 2:**
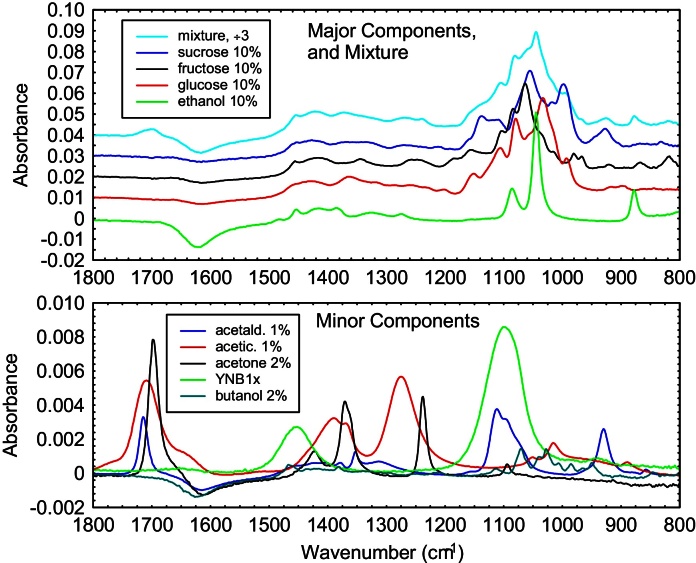
Spectra for the components and the artificial mixture. Note the scale change in absorbance for major *vs*. minor components. The mixture (÷3) and the sugars have been offset in absorbance steps of 0.01, for clarity. The absorbances are relative to water as the background.

Our first verification of the methodology was to make an ideal FTIR spectrum for an artificial “mixture” by adding FTIR spectra from nine aqueous components, with appropriate multipliers. The components were sucrose, glucose, fructose, YNB, ethanol, butanol, acetone, acetaldehyde, and acetic acid. The percentages are representative of a partially complete fermentation of mixed sugars by microorganisms. For this verification we assumed the component spectra were perfect, giving a perfect FTIR spectrum for the “mixture.” The spectra are shown in Fig. 2.

Not surprisingly, our computer program based on MPLM gave an exact fit. To simulate a real-life sample spectrum, we added a random value from a normal distribution (of magnitude 0.5% and centered on zero) to each absorbance value in the ideal spectrum of the mixture. This would be considered a noisy measurement. Sample spectra with 0.1% random error in the absorbance appeared most similar to good experimental spectra in our research. (Component spectra can be repeatedly measured and averaged, until their noise is not an issue compared to sample spectra noise.) To save some time, the first-pass boundaries of the fits were from 0 to 12, with a stepsize of 3. (This ensured that none of the correct component values were directly on any of the multipass grids.) The RMS error for all fits was 0.00042(1). The computational time was 1600 s per fit. The component concentrations thereby obtained, for five runs, are shown in Table 1. It is clear that averaging a few runs allows MPLM to give relative errors of 2% or less for all components, even with complicated mixtures, component contributions differing by up to 50×, and large levels of “measurement” noise. The absolute accuracy for all component percentages was better than our target accuracy of ±0.1%.

### Verification with a yeast fermentation mixture

Our second verification of the methodology used real mixture data, fitting the FTIR spectrum of a sample from a bio-hybrid fuel cell. The sample was a yeast fermentation of glucose, which had been run for some time in a direct ethanol fuel cell. It was expected to contain ethanol, glucose, acetic acid, yeast extract (Y), and bacterial peptone (P). The methodology was challenged by either omitting one or two components from the fit, or by adding additional components. We evaluated the goodness of the MPLM fit primarily using *ɛ*_*RMS*_ from (2). We also used the slope (*a*), *y*-intercept (*b*), and correlation coefficient (*R*^2^) of a straight-line (*y* = *ax* + *b*) fit between the measured (*y*) and computed (*x*) FTIR absorbances, where the computed absorbances were obtained from (1) using the fitted values for *C*_*n*_ and the known $A_{n,m}^{\text{Component}}$. Ideally *ɛ*_*RMS*_ = 0, and the straight-line fit would yield *a* = 1, *b* = 0, and *R*^2^ = 1. The results are shown in Table 2, Table 3. (*R*^2^ and *a* are subtracted from 1 so that smaller numbers indicate a better fit in all four statistics columns.)

If a high-concentration component (*e.g.*, ethanol, glucose) is omitted, a cursory visual comparison of the fit spectrum compared to the sample spectrum is sufficient to determine that an important component has been left out. The statistical fit indicators in Table 2 are just as clear that something is amiss. The fitted component concentrations are not even close to correct. On the other hand, if a low-concentration but non-trivial component (*e.g.*, acetic acid, yeast extract, peptones) is omitted, the overall quality of the fit still looks good to the eye. The fitted *C*_*n*_ of the major components barely change, so accidentally omitting a minor component doesn’t entirely invalidate the QA. However, the statistics are clearly worse.

Sometimes the computed concentration of a component can be changed significantly if its spectrum is similar enough to the omitted spectrum. The cases for omitting yeast extract or peptone in Table 2 show that either one alone can imitate the other, especially at low concentrations. Only slight errors in the other component concentrations are engendered, and there are only small statistical indications of a problem. However, omitting both (*i.e.*, the Y + P case in Table 2) causes large errors in the other component concentrations and is clearly flagged as a mistake by all four statistical indicators.

Adding either sucrose or fructose improved the fit slightly, according to all four statistical indicators (Table 3). The fitted concentrations of ethanol, acetic acid, yeast extract, and peptone remained almost unchanged. The fitted concentration of glucose decreased slightly, almost exactly compensated for by the increase (from assumed zero) of the sucrose or fructose. The fitted concentration was 0.18% (0.15%) for sucrose (fructose), for a consistent total sugar concentration of 5.33% (5.36%). Including both sucrose and fructose in the fit gave results almost identical to sucrose only. Per the manufacturer's analysis, our glucose was in fact only 99.8% glucose. The MPLM fit found non-zero concentrations of sucrose probably because it was actually in the sample, originally as a trace contaminant in the glucose, and becoming more noticeable after the yeast had preferentially catabolized glucose.

This result is in stark contrast to the results obtained by including galactose, methanol, or propanol as a component. There was no reason to expect any of them in our sample. Although their FTIR spectra share some features with spectra of known components (*i.e.*, other sugars and ethanol), the MPLM was not tricked, regardless of the combinations tried. It consistently gave zero for their concentrations.

Despite the successes, the statistical indicators were larger than expected, which implied there was still a missing (trace) component. Adding both acetone and butanol as possible components improved the fit slightly, with 0.14% acetone and 0.15% butanol. See Table 3. Their presence would be consistent with contamination by *Clostridium acetobutylicum*, another organism in our lab. However, the 1:1 ratio of acetone to butanol does not match what would be expected. We hypothesized that the tubing we used was leaching plasticizer into the fermentate, due to the high ethanol level. Commonly-used plasticizers have some molecular structures that are similar to those of acetone and butanol. By soaking the tubing in ethanol, we obtained the leachate, and its FTIR spectrum. It was added as a possible component to the fit. Again, the resulting percentages of the other (non-trace) components were scarcely changed, but the overall fit was noticeably improved. See Table 3. The best fit was obtained with 0.15% sucrose, 0.01% fructose, and 0.87% leachate. The spectrum of the leachate (not shown) was similar to dibutyl phthalate, which is indeed a commonly used plasticizer. Thus, the MPLM was sensitive enough to indicate a potential problem with our setup while it was still a minor problem.

## Example applications of the methodology to bio-hybrid fuel cell research

We now briefly present several ways we have applied the MPLM for FTIR in our bio-hybrid fuel cell research, as illustrative examples of its utility and sensitivity.

### Tracking cultures of microorganisms

Cultures of bacteria or fungi can be difficult to track with most analysis methods, because the component concentrations change dramatically, often in opposite directions. FTIR, however, can handle high and low concentrations simultaneously. In a research environment, many variables of the cultures can change, such as organisms, media compositions, temperatures, oxygenation, stirring, and resulting products.

**Fig. 3:**
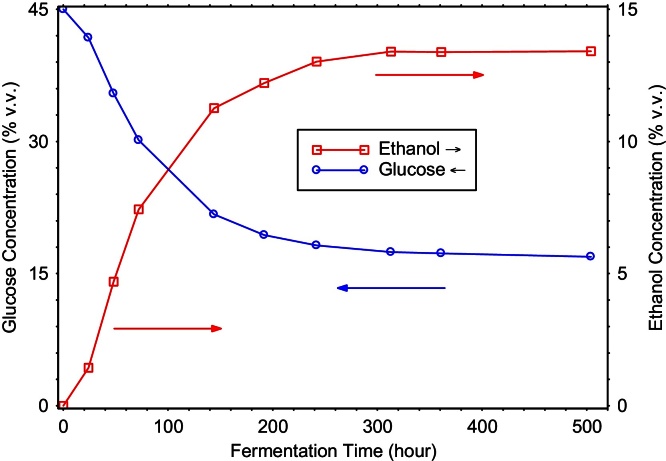
During a fermentation of glucose by yeast, the falling/rising levels of glucose/ethanol can be easily tracked.

Fig. 3 shows a typical (simple) example, an anaerobic fermentation by yeast (*Saccharomyces cerevisiae*) of a syrupy solution of 45% glucose in water plus some trace nutrients. After 500 h, the glucose level has dropped by more than half, while the ethanol level has climbed from zero to almost 14%. (The other media components are nearly steady and are not shown.)

### Tracking fuel cell anode reactions

**Fig. 4:**
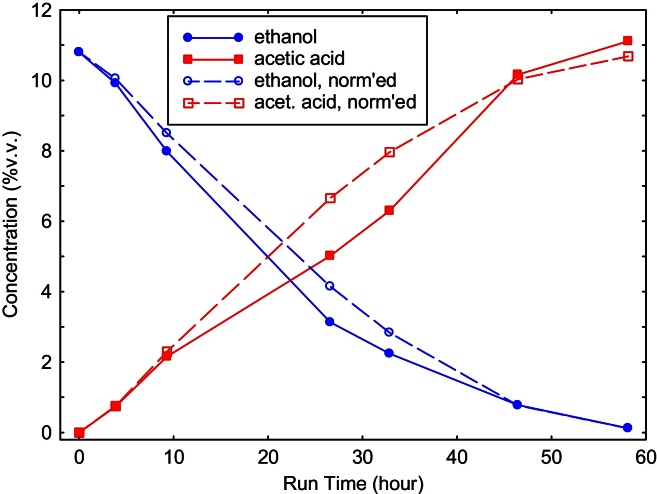
During a direct ethanol fuel cell run, the falling/rising levels of ethanol/acetic acid can be easily tracked. The normalized values are relative concentrations, to account for the reduced total volume due to evaporation.

In a bio-hybrid fuel cell, the microbes provide a fuel (*e.g.*, ethanol) that powers a conventional fuel cell. In Fig. 4, we show results from the MPLM, tracking a run in a direct ethanol fuel cell (DEFC). Even in the presence of media components (not shown), the falling/rising levels of ethanol/acetic acid in the anode compartment of the DEFC can be accurately ascertained. (The normalized values are relative concentrations, to account for evaporative losses.)

### Tracking protonation state as a pH indicator

Directly determining the pH of solutions by using FTIR spectra for H_3_O^+^ and OH^−^ would be a very difficult measurement, since the concentrations of those ions are low, especially in nearly-neutral solutions. This presents a problem for precisely tracking pH in bio-hybrid fuel cells using FTIR. Fortunately, in our research, pH changes occur mostly due to production of acetic acid. The acetate ion exists in either a protonated (CH_3_COOH) or deprotonated (CH_3_COO^−^) state, and the two states have different FTIR peaks. Treating the two states as separate components, and tracking the concentration of each state, allows us to determine the pH, either through a calibration measurement (as below) or through the Henderson-Hasselbach equation. Alternatively, one could use the method in reverse to measure p*K*_a_ [12].

**Fig. 5:**
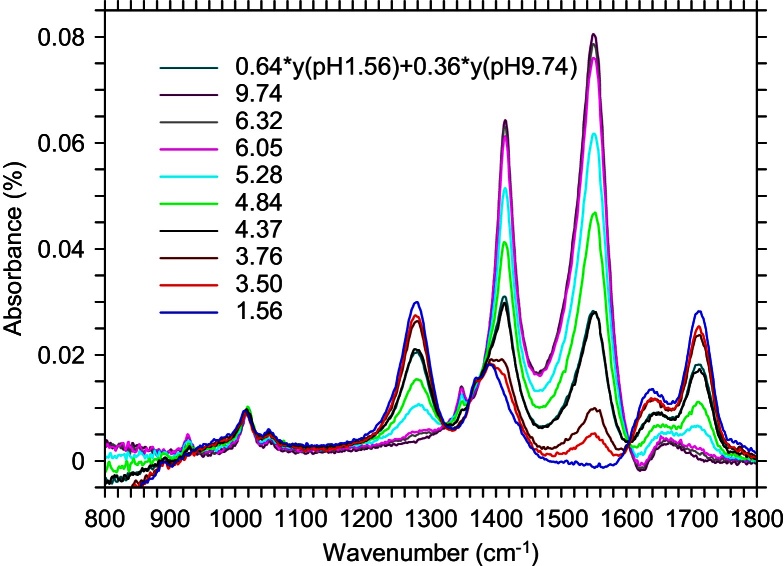
Spectra for each titration step, labeled with the measured pH. Each intermediate spectrum can be fit as a linear combination of the spectra at pH 1.56 and pH 9.74. Also shown is an example fit for pH 4.37. It can be seen that the correspondence is excellent.

**Fig. 6:**
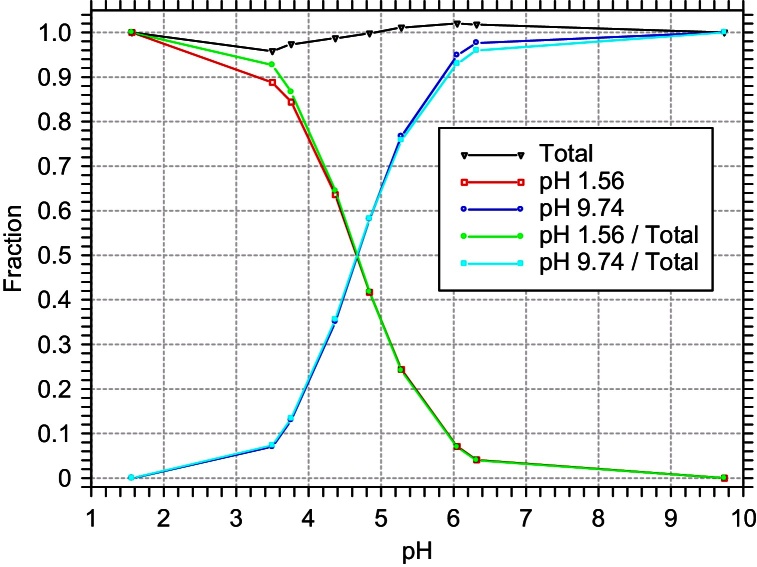
The relative fraction of protonated (pH 1.56) and deprotonated (pH 9.74) acetate ions in each titration sample *vs.* measured pH. The relative fraction is either taken directly from the fits (red and blue lines), or is normalized to the total fraction in the fits (green and cyan lines). The total fraction from the fits is also shown (black line).

We prepared a solution of 1 M sodium acetate (pH 9.74) and titrated it with 12.1 M hydrochloric acid, measuring the pH and taking an FTIR spectrum at each titration step. At intermediate pH values, where the solution is a mixture of the CH_3_COOH and CH_3_COO^−^ states, the spectrum can be fit as a linear combination of the spectra for the two end points of the titration, where the acetate ion is almost entirely either protonated (pH 1.56) or deprotonated (pH 9.74). Spectra for each titration step are given in Fig. 5, labeled with the measured pH, along with a sample fit for pH 4.37. The relative fraction of each spectrum *vs.* measured pH is given in Fig. 6. Because of slight density changes, the fraction totals at intermediate pH did not add up to exactly 1.0. The correction for this is also shown in Fig. 6.

### Measuring diffusion across membranes

**Fig. 7:**
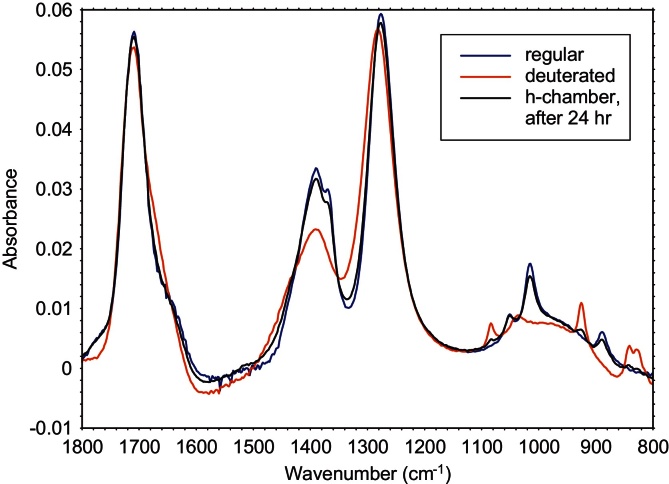
FTIR spectra of regular (h-) and deuterated (d-) 10% acetic acid, plus a spectrum of a sample from the h-chamber after 24 h of diffusion. The sample spectrum (h-chamber, after 24 h) has begun to show deuterated characteristics.

Diffusion across a reverse osmosis (RO) membrane was measured by placing two 10% (v/v) acetic acid solutions into two chambers separated by an RO membrane. The two solutions were distinguished by using regular acetic acid in one chamber (labeled the h chamber) and acetic acid where the methyl group was deuterated in the other (labeled the d chamber) This setup was used to eliminate osmotic pressure effects that would complicate the interpretation of the data if the acetic acid chamber was separated from a completely aqueous chamber by the RO membrane. After setup, the system was then allowed to equilibrate by diffusion of both forms of acetic acid across the RO membrane. Small samples were taken from both chambers twice per day for three days. As shown in Fig. 7, the FTIR spectrum of d-acetic acid has peaks that are slightly shifted (by about 10 cm^−1^) from those for h-acetic acid.

**Fig. 8:**
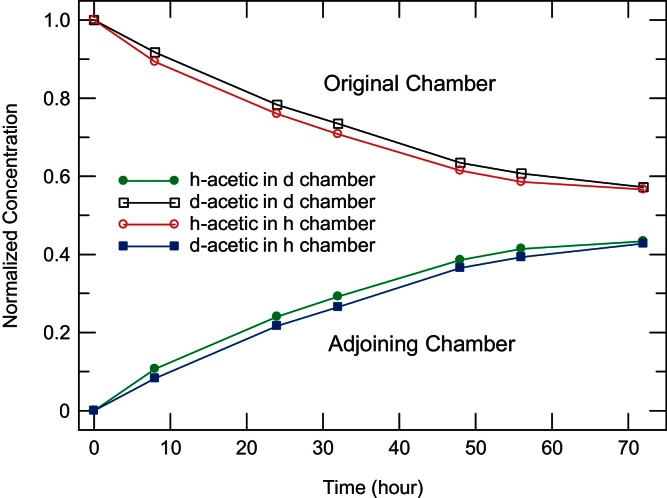
Even small concentrations of very similar molecules can be accurately tracked, as in this experiment to measure the diffusion of acetic acid across a membrane. Initially the h (d) chamber contains 10% regular (deuterated) acetic acid. It is clear that diffusion of deuterated molecules is slightly slower.

Comparison with Fig. 5 spectra indicates that the 10% acetic acid (being a weak acid) is essentially completely protonated. Despite the small spectral differences, the MPLM can accurately analyze the mixtures with no difficulty, to obtain the concentrations shown in Fig. 8. The filled circles (squares) in Fig. 8 show the normalized concentration of h-acetic (d-acetic) acid in the d (h) chamber where it was initially absent, while the open circles (squares) show the h-acetic (d-acetic) concentration in the h (d) chamber where it was initially 10%. (The initial concentrations are normalized to 1 in the graph.) The deconvoluted data obtained using the methodology can be fit using Fick's First Law [13] to calculate a membrane permeability of 2.7 cm/s for regular h-acetic acid. Not only does the data fit well to theory, but the accuracy and precision of the methodology also enables one to determine that the d-acetic acid diffuses slightly more slowly, as expected (permeability of 2.5 cm/s).

## Conclusion

We have described the MPLM in terms of FTIR, since we have only used it for FTIR. However, we can think of no intrinsic reason why the MPLM couldn’t be used for UV–vis spectroscopy, Raman, SERS, X-ray reflectance, or any experimental method in which the spectrum from a mixture is a linear combination of the spectra from the constituents. Moreover, the MPLM could be trivially extended to combinations of complementary measurements (*e.g.*, FTIR + Raman), allowing for greater discrimination in mixtures of similar components. It could also of course be restricted to only sections of the spectra with sufficient signal-to-noise ratio.

## Figures and Tables

**Table 1:** Fit results for artificial mixture's noisy spectra, with correct values at top. There are five runs of 0.5% noise since the randomness of the noise affected the fit results. Note also that spectra with 0.1% random error appeared most similar to typical experimental spectra in our research. The spectra with 0.5% error used here would be considered noisy.

	Sucrose	Glucose	Fructose	YNB	Ethanol	Butanol	Acetone	Acetald.	Ac.acid
Exact	10.000	10.000	10.000	1.000	10.000	2.000	2.000	1.000	1.000
#1	9.996	9.999	10.038	0.984	9.984	2.008	1.999	1.014	1.008
#2	9.972	10.017	10.013	1.007	9.973	1.950	1.989	1.019	0.997
#3	9.977	10.056	10.019	0.978	9.938	1.969	2.042	1.035	0.983
#4	9.987	10.017	10.011	0.990	10.026	1.888	1.990	1.026	0.993
#5	10.051	9.958	10.016	0.992	10.003	1.965	2.006	0.980	0.992
avg	9.997	10.009	10.019	0.990	9.985	1.956	2.005	1.015	0.995
*σ*_*n*_	0.028	0.032	0.010	0.010	0.030	0.039	0.019	0.019	0.008
*e*_rel_	−0.03%	0.09%	0.19%	−1.0%	−0.15%	−2.2%	0.25%	1.5%	−0.50%

**Table 2:** Effect on the MPLM fit of omitting components, using FTIR data from a real sample.

Omitted Cmpnt.	ethl. (%)	gluc. (%)	ac.ac. (%)	yst.ex. (%)	pep. (%)	Y + P (%)	*ɛ*_*RMS*_ (%)	1 − *R*^2^ (×10^−4^)	1 − *a* (×10^−4^)	*b* (×10^−5^)
None	9.52	5.31	0.34	1.04	1.34	2.38	.0569	30	34	7.9
Ethanol	–	9.23	0.34	0.21	0.00	0.21	.4498	1872	−167	−32
Glucose	15.4	–	0.76	4.35	0.00	4.35	.4091	1468	480	110
Acetic acid	9.54	5.44	–	0.98	1.58	2.56	.0792	52	130	31
Yeast extr.	9.57	5.45	0.33	–	2.34	2.34	.0633	36	60	14
Peptone	9.36	5.18	0.36	2.31	–	2.31	.0671	41	26	6.1
Y + P	7.28	6.54	0.77	–	–	–	.2299	380	480	120

**Table 3:** Effect on the MPLM fit of adding components, using FTIR data from a real sample.

Added Cmpnt.	ethl. (%)	gluc. (%)	ac.ac. (%)	yst.ex. (%)	pep. (%)	Y + P (%)	*ɛ*_*RMS*_ (%)	1 − *R*^2^ (×10^−4^)	1 − *a* (×10^−4^)	*b* (×10^−5^)
None	9.52	5.31	0.34	1.04	1.34	2.38	.0569	30	34	7.9
Sucrose	9.54	5.15	0.34	0.99	1.39	2.38	.0564	29	27	6.4
Fructose	9.52	5.21	0.33	0.95	1.41	2.36	.0566	29	30	6.9
Adding galactose, methanol, or propanol as possible new components made no difference in the fits for the known components, and gave 0% for the new components										
Butanol + acetone	9.58	5.23	0.31	1.07	1.37	2.44	.0567	24.5	29.2	9.0
Leachate (0.87%)	9.61	5.23	0.23	0.97	1.53	2.51	.0543	27	34	8.3
Sugars + leachate	9.61	5.11	0.23	0.89	1.61	2.50	.0539	30	27	6.9

## References

[1] Nguele R., Al-Salim H.S., Mohammad K. (2014). Modeling and forecasting of depletion of additives in car engine oils using attenuated total reflectance fast transform infrared spectroscopy. Lubricants.

[2] Kiefer J. (2015). Recent advances in the characterization of gaseous and liquid fuels by vibrational spectroscopy. Energies.

[3] Kumar S., Barth A. (2010). Following enzyme activity with infrared spectroscopy. Sensors.

[4] Mueller D., Ferrão M.F., Marder L., da Costa A.B., de Cássia de Souza Schneider R. (2013). Fourier transform infrared spectroscopy (FTIR) and multivariate analysis for identification of different vegetable oils used in biodiesel production. Sensors.

[5] Kačuráková M., Capeka P., Sasinková V., Wellnerb N., Ebringerová A. (2000). FT-IR study of plant cell wall model compounds: pectic polysaccharides and hemicelluloses. Carbohydr. Polym..

[6] Mackie D.M. (2012). Biometrics via IR spectroscopy of the epidermis: potential and difficulties. Proc. SPIE 8371, Sensing Technologies for Global Health, Military Medicine, Disaster Response, and Environmental Monitoring II; and Biometric Technology for Human Identification IX, 83711T.

[7] Holthoff E., Bender J., Pellegrino P., Fisher A. (2010). Quantum cascade laser-based photoacoustic spectroscopy for trace vapor detection and molecular discrimination. Sensors.

[8] Mariey L., Signolle J.P., Amiel C., Travert J. (2001). Discrimination, classification, identification of microorganisms using FTIR spectroscopy and chemometrics. Vib. Spectrosc..

[9] Grube M., Gapes J.R., Schuster K.C. (2002). Application of quantitative IR spectral analysis of bacterial cells to acetone–butanol–ethanol fermentation monitoring. Anal. Chim. Acta.

[10] Baker M.J., Trevisan J., Bassan P., Bhargava R., Butler H.J., Dorling K.M., Fielden P.R., Fogarty S.W., Fullwood N.J., Heys K.A., Hughes C., Lasch P., Martin-Hirsch P.L., Obinaju B., Sockalingum G.D., Sulé-Suso J., Strong R.J., Walsh M.J., Wood B.R., Gardner P., Martin F.L. (2014). Using Fourier transform IR spectroscopy to analyze biological materials. Nat. Protoc..

[11] Berger M.J., Colella P. (1989). Local adaptive mesh refinement for shock hydrodynamics. J. Comput. Phys..

[12] Allen R.I., Box K.J., Comer J.E.A., Peake C., Tam K.Y. (1998). Multiwavelength spectrophotometric determination of acid dissociation constants of ionizable drugs. J. Pharm. Biomed. Anal..

[13] Roque-Malherbe R.M.A. (2010). Physical Chemistry of Materials – Energy and Environmental Applications. http://app.knovel.com/.

