# Supplementary material for: Simple, fast, and accurate methodology for quantitative analysis using Fourier transform infrared spectroscopy, with bio-hybrid fuel cell examples
Source: MethodsX. 2016 Feb 21;3:128–38. doi: 10.1016/j.mex.2016.02.002 (PMC4781924; doi:10.1016/j.mex.2016.02.002)
Supplement: Supplementary file 1 [file mmc1.docx]

' At this point, A contains all the chosen component spectra and the sample spectrum,

' with all of them in the same order and all for the same wavenumbers.

‘ Note that NextRngMult, RngDivide, and RngDivMin are set elsewhere, by the GUI.

‘ The values we used were 0.8, 4.0, and 0.001, respectively.

'MAIN LOOP START: nested FOR loops find the best fit (least SQRerror)

Dim SQRerror, SQRerrorLEAST As Double

SQRerrorLEAST = 100000000.0

Dim Cpct0, Cpct1, Cpct2, Cpct3, Cpct4, Cpct5, Cpct6, Cpct7, Cpct8, CpctBEST(8) As Double

Dim iWstep, iPassMAX As Integer

If tbSkipData.Text > 0 Then

iWstep = tbSkipData.Text + 1

Else

iWstep = 1

End If

iPassMAX = 9 : iPassMAX = Math.Max(iPassMAX, 1) ‘ Ignore second statement; It is vestigial error code.

For iPass As Integer = 1 To iPassMAX

ProgressBar1.Maximum = CMaxData(8) * 100

ProgressBar1.Value = 0

If cbMultiPass.Checked Then

labelMultiPass.Text = "Lvl: " + Str(iPass)

Me.Update()

End If

For Cpct8 = CMinData(8) To CMaxData(8) Step CStepData(8)

For Cpct7 = CMinData(7) To CMaxData(7) Step CStepData(7)

For Cpct6 = CMinData(6) To CMaxData(6) Step CStepData(6)

For Cpct5 = CMinData(5) To CMaxData(5) Step CStepData(5)

For Cpct4 = CMinData(4) To CMaxData(4) Step CStepData(4)

For Cpct3 = CMinData(3) To CMaxData(3) Step CStepData(3)

For Cpct2 = CMinData(2) To CMaxData(2) Step CStepData(2)

For Cpct1 = CMinData(1) To CMaxData(1) Step CStepData(1)

For Cpct0 = CMinData(0) To CMaxData(0) Step CStepData(0)

SQRerror = 0.0

For iW = 0 To iWend Step iWstep

SQRerror = SQRerror + (Cpct0 * A(0, iW) + Cpct1 * A(1, iW) + _

Cpct2 * A(2, iW) + Cpct3 * A(3, iW) + _

Cpct4 * A(4, iW) + Cpct5 * A(5, iW) + _

Cpct6 * A(6, iW) + Cpct7 * A(7, iW) + _

Cpct8 * A(8, iW) - _

A(9, iW)) ^ 2

Next

If SQRerror < SQRerrorLEAST Then

SQRerrorLEAST = SQRerror

CpctBEST = {Cpct0, Cpct1, Cpct2, Cpct3, Cpct4, Cpct5, Cpct6, Cpct7, Cpct8}

End If

Next

Next

Next

Next

Next

Next

Next

Next

ProgressBar1.Value = Cpct8 * 100

Me.Update()

Next

If (cbMultiPass.Checked And iPass < iPassMAX) Then 'change min,max,step based on results so far

Dim LastPass As Boolean = True 'usually this gets changed to False in the For loop below

For iii As Integer = 0 To iA

CMinData(iii) = Math.Max((CpctBEST(iii) - NextRngMult * CStepData(iii)), 0.0)

CMaxData(iii) = Math.Min(CpctBEST(iii) + NextRngMult * CStepData(iii), 100.0)

CStepData(iii) = Math.Max((CMaxData(iii) - CMinData(iii)) / RngDivide, RngDivMin)

If (CStepData(iii) <> 0.001) Then LastPass = False

Next

If LastPass Then iPass = iPassMAX - 1

iA = -1 'Write the fit parameters to the appropriate boxes

For iComp = 0 To 12

If SpecComps(iComp).cbUse.Checked Then

iA = iA + 1

SpecComps(iComp).tbFit.Text = CpctBEST(iA)

Else

SpecComps(iComp).tbFit.Text = 0.0

End If

Next

Me.Update()

Else

Exit For

End If

Next

'MAIN LOOP END: nested FOR loops find the best fit
